# Delayed-onset parkinsonism possibly associated with elranatamab treatment for relapsed/refractory multiple myeloma: a case report of a poorly characterized neurological event

**DOI:** 10.1007/s00277-026-07163-1

**Published:** 2026-07-07

**Authors:** Asuka Kono, Yoshihiro Umezawa, Kensuke Oshima, Keisuke Tanaka, Yuki Kobayashi, Takehiko Mori, Masahide Yamamoto

**Affiliations:** 1 Department of Hematology, Graduate School of Medical and Dental Sciences, Institute of Science Tokyo, 1-5-45 Yushima, Bunkyo-Ku, Tokyo, 113-8519 Japan; 2 Department of Neurology and Neurological Science, Graduate School of Medical and Dental Sciences, Institute of Science Tokyo, Tokyo, Japan

**Keywords:** Multiple myeloma, Parkinsonism, Movement and neurocognitive toxicity (MNT), B-cell maturation antigen (BCMA), Bispecific antibodies (BsAbs)

## Abstract

T cell–redirecting therapies directed against B-cell maturation antigen (BCMA) have revolutionized the treatment of relapsed or refractory multiple myeloma (MM). BCMA-directed CAR-T cell therapy has been reported to cause a parkinsonism-predominant neurotoxicity referred to as movement and neurocognitive toxicity (MNT). MNT is considered distinct from immune effector cell-associated neurotoxicity syndrome (ICANS) in terms of its clinical features and time to onset. However, MNT-like delayed-onset parkinsonism has not been well documented after BCMA-directed bispecific antibody (BsAb) therapy. We report the case of a woman with relapsed/refractory MM who developed parkinsonism following elranatamab. She experienced neither cytokine release syndrome nor ICANS. Beginning the day after the Week 7 dose, she developed bradykinesia, repeated falls, gait disturbance, and cognitive decline. Neurological examination revealed hypomimia, bradykinesia, upper extremity rigidity, postural instability, and a shuffling gait, consistent with parkinsonism. Brain MRI and dopamine transporter single-photon emission computed tomography findings showed no findings suggestive of degenerative Parkinson’s disease. Her symptoms gradually improved after discontinuation of elranatamab. This case highlights the possibility of delayed-onset parkinsonism with neurocognitive symptoms following treatment with a BCMA-directed BsAb. Clinicians should recognize the potential for delayed-onset parkinsonism and ensure close neurological monitoring in patients receiving these agents.

## Introduction

Multiple myeloma (MM) remains incurable and is characterized by frequent relapses and the eventual development of treatment-refractory disease. In recent years, therapeutic advances—particularly combination regimens incorporating proteasome inhibitors (PIs), immunomodulatory drugs (IMiDs), and anti-CD38 monoclonal antibodies (mAbs)—have significantly improved patient outcomes [1]. Nevertheless, patients with triple-class refractory disease continue to have a dismal prognosis [2]. For these patients, T cell–redirecting therapies, including chimeric antigen receptor (CAR)-T cell therapy and bispecific antibodies (BsAbs), have emerged as effective treatment options, and agents targeting B-cell maturation antigen (BCMA) are now widely used [3–7].

T cell–redirecting therapies are associated with characteristic adverse events, such as cytokine release syndrome (CRS) and neurotoxicity. In pivotal trials of BCMA-directed CAR-T cell therapy, grade ≥ 3 neurotoxicity has been reported in approximately 20% of patients [3, 4], whereas BsAbs have shown a lower incidence of grade ≥ 3 neurotoxicity, at around 3–5% [5–7]. Immune effector cell–associated neurotoxicity syndrome (ICANS) is the most common form of neurotoxicity, typically developing within approximately 1–10 days after CAR-T cell therapy or BsAb administration and often occurring following CRS [3–7]. The spectrum of symptoms ranges from delirium and language impairments to seizures and coma. Cognitive dysfunction is the most frequently observed symptom, and it typically resolves with appropriate treatment, including steroid administration [8]. However, a delayed-onset neurotoxicity characterized by parkinsonism—termed movement and neurocognitive toxicity (MNT)—has been increasingly recognized as an important complication in the context of BCMA-directed CAR-T cell therapy. We describe herein a case of delayed-onset parkinsonism with neurocognitive symptoms resembling MNT that occurred during treatment with elranatamab, a BCMA-directed BsAb.

## Case

A 69-year-old woman was diagnosed with multiple myeloma (IgG-κ type; International Staging System: stage II) in January 2017. At diagnosis, multiple vertebral fractures were present. Fluorescence in situ hybridization (FISH) analysis revealed high-risk cytogenetic abnormalities, including deletion 17p and t (4;14).

Initial therapy with the VRd regimen, consisting of bortezomib (BOR), lenalidomide (LEN), and dexamethasone (DEX), was initiated in February 2017. However, due to the development of a skin rash, the regimen was changed to IRd therapy consisting of ixazomib, LEN, and DEX. The patient achieved a stringent complete response (CR), and maintenance therapy with lenalidomide was continued thereafter for 12 months.

In March 2022, approximately two years after treatment discontinuation, M-protein became detectable by immunofixation, and relapse was diagnosed. DRd therapy consisting of daratumumab, LEN, and DEX was initiated, resulting in a return to stringent CR. After 33 cycles of DRd therapy, relapse with rapid disease progression was observed.

**Fig. 1:**
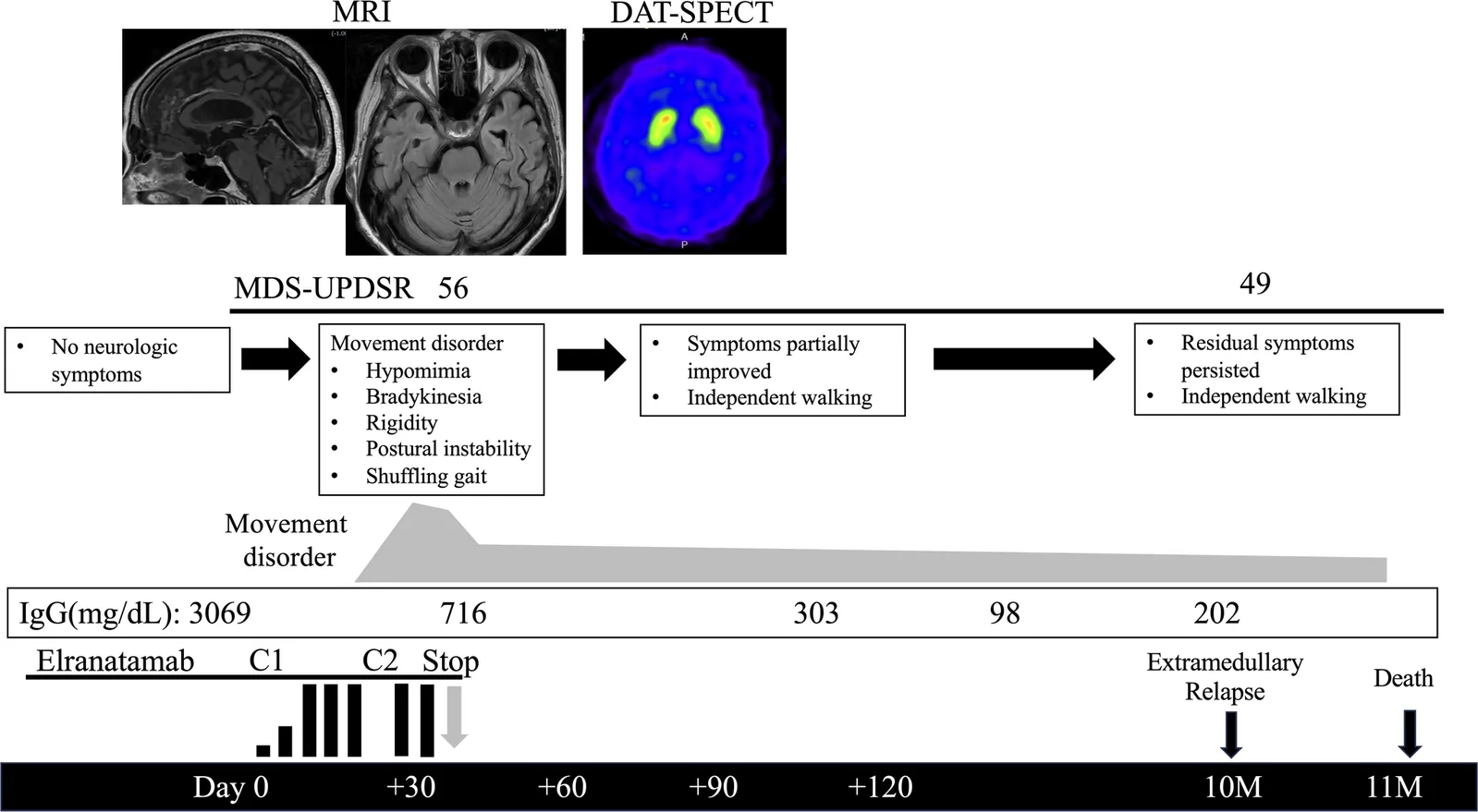
Clinical course, neurological findings, and neuroimaging after elranatamab treatment. The upper panel shows representative neuroimaging findings arranged according to the clinical course. Brain MRI demonstrated mild hippocampal atrophy without other clinically significant abnormalities on sagittal T1-weighted imaging (left) and axial fluid-attenuated inversion recovery imaging (FLAIR; right). Dopamine transporter single-photon emission computed tomography (DAT-SPECT) with 123I-ioflupane showed preserved dopamine transporter uptake in the basal ganglia. The lower panel shows the clinical course of neurological symptoms after elranatamab administration

Given the triple-class refractory disease, elranatamab, a BCMA-directed BsAb, was initiated in December 2024. The clinical course after elranatamab initiation is shown in Fig. 1. The initial cycle was conducted under inpatient management, during which the clinical course was uneventful without CRS or ICANS, and the patient was discharged. Elranatamab was effective, and serum IgG levels decreased from 3069 mg/dL to 716 mg/dL. Elranatamab was continued once weekly. After the Week 7 dose, the patient exhibited bradykinesia and experienced repeated falls. Gait disturbance and cognitive impairment were also observed. Accordingly, the Week 8 dose was withheld, and the patient was admitted to the hospital.

Neurological evaluation revealed hypomimia, bradykinesia, rigidity of the upper extremities, postural instability, and a shuffling gait, all of which were consistent with parkinsonism. Motor strength, reflexes, and sensation were intact. The patient scored 56 points on the Movement Disorder Society–Unified Parkinson’s Disease Rating Scale (MDS-UPDRS) Part III [9], which assesses the severity of motor symptoms in Parkinson’s disease. In addition, the score on the Hasegawa Dementia Scale–Revised (HDS-R), which assesses cognitive function [10], was 14. Brain CT and magnetic resonance imaging (MRI; Fig. 1) revealed only mild hippocampal atrophy without abnormalities in the basal ganglia, cerebellum, or brainstem, including the midbrain tegmentum, and without evidence of cerebrovascular disease or intracranial tumors. Levodopa was not effective for her symptoms. Dopamine transporter single-photon emission computed tomography (DAT-SPECT) with 123I-ioflupane demonstrated normal uptake in the striatum, including the putamen and caudate nucleus (Fig. 1). *Based on these* *findings*, *degenerative* *Parkinson’s disease was considered* *unlikely*, *and a drug-related etiology* *was suspected.*

Since the neurological symptoms emerged after the initiation of elranatamab, and there was no history of prior exposure to neuroleptics or other dopamine antagonists, a possible association with elranatamab was considered.

After discontinuation of elranatamab, her parkinsonian symptoms, including hypomimia, extremity rigidity, gait disturbance, and cognitive impairment, gradually improved over two weeks without any treatment. Three weeks after discontinuation of elranatamab, she regained the ability to walk with a walking aid and was discharged. Thereafter, her neurological symptoms partly persisted, but the MDS-UPDRS score improved from 56 to 49. Her gait improved sufficiently for her to walk independently in 14 weeks after elranatamab discontinuation. Despite discontinuation, elranatamab led to a very good partial response (VGPR) in MM. Eight months after elranatamab discontinuation, an extramedullary relapse occurred. The patient declined further treatment and was subsequently transitioned to palliative care.

## Discussion

Neurological adverse events are known to occur with T-cell redirecting therapies. ICANS, an early-onset neurotoxicity of the central nervous system (CNS) that may manifest as altered consciousness or seizures, has been reported following both CAR-T cell therapy and BsAb treatment. Furthermore, neurological toxicities that differ from ICANS in terms of clinical features, onset pattern, and timing have increasingly been reported [11].

Delayed-onset parkinsonism with neurocognitive symptoms has been described mainly after BCMA-directed CAR-T cell therapy, particularly ciltacabtagene autoleucel. These cases share common characteristics, including delayed onset several weeks after treatment and the development of motor disturbances and cognitive impairment. Because these features differ from those of ICANS, the condition has recently been referred to as movement and neurocognitive toxicity (MNT) [12–14].

Neurological adverse events associated with BCMA-directed BsAbs have generally been reported as ICANS or nonspecific neurological toxicities, including headache, encephalopathy, motor dysfunction, and sensory neuropathy. In the pivotal phase 2 MagnetisMM-3 study of elranatamab, ICANS occurred in 4 of 119 patients (3.4%), and all events were grade 1 or 2. Motor dysfunction and sensory neuropathy were reported in 17.1% and 13.8% of patients, respectively, whereas Parkinson-like events were not observed [5]. In pivotal studies of other BCMA-directed BsAbs, including teclistamab and linvoseltamab, ICANS was also reported in a minority of patients and, when timing was described, generally occurred early during step-up dosing or in temporal proximity to CRS [6, 7]. These findings suggest that neurological toxicity associated with BCMA-directed BsAbs has usually been characterized as early-onset ICANS or nonspecific neurological events.

 In contrast, reports of delayed or non-acute neurological disorders after BsAb therapy for multiple myeloma remain limited. Lutfi et al. reported two cases of persistent cognitive impairment following teclistamab treatment, which were described as an unusual, prolonged form of ICANS [15]. Parkinsonism was not described in either case. Although talquetamab targets GPRC5D rather than BCMA, delayed or non-acute neurological events such as dizziness, ataxia, and balance disorders have been reported with talquetamab treatment [16–18]. In addition, progressive multifocal leukoencephalopathy (PML) has been reported after prolonged exposure to BsAb therapy [19, 20]. Although PML represents an opportunistic CNS infection rather than direct drug-induced neurotoxicity, it should be considered in the differential diagnosis of delayed neurological symptoms during long-term BsAb treatment. These published reports and publicly available safety data are summarized in Table 1. Well-documented clinical reports of parkinsonism associated with BCMA-directed BsAb therapy are extremely limited. A search of the Japanese Adverse Drug Event Report database (JADER), maintained by the Pharmaceuticals and Medical Devices Agency (PMDA), identified one spontaneous suspected adverse drug reaction report of parkinsonism for elranatamab [21], whereas no such reports were identified for teclistamab or talquetamab under the same search conditions. Because detailed clinical information was unavailable, this report could not be used to characterize the timing or clinical features of the event.

**Table 1:** Published reports and publicly available safety data on delayed or non-acute neurological events and CNS complications during bispecific antibody therapy for multiple myeloma

No	Therapy	Target	Source type	Neurological events / CNS complications	Onset from therapy initiation	Comments
1 [15]	Teclistamab	BCMA × CD3	Case reports	Cognitive impairment	Reported in two cases: symptoms persisted over Day 8–18 and Day 1–31, respectively.	Unusual, prolonged form of ICANS
2 [16]	Talquetamab	GPRC5D × CD3	Case series	Dizziness, ataxia, and balance disorders	Reported in four patients; onset: Day 21, Day 24, Cycle 3 Day 10, and Cycle 2 Day 10.	Non-ICANS neurological events with delayed or variable onset.
3 [17, 18]	Talquetamab	GPRC5D × CD3	MonumenTAL-1 safety analysis / public safety information	Dizziness, ataxia, and balance disorders	Late-onset ataxia was reported in 4.1%; median onset was 77 days (range, 2–463) from first dose.	Non-ICANS neurological events with delayed or variable onset.
4 [19, 20]	BCMA-directed BsAbs	BCMA × CD3	Case reports / literature review	PML*	Delayed onset; median onset of 22 months in a case series	CNS infectious complication during ongoing BsAb therapy.
5 [21]	Elranatamab	BCMA × CD3	Public pharmacovigilance database	Parkinsonism	Not available	One suspected adverse drug reaction report of parkinsonism was identified in PMDA/JADER^**^.
6	Elranatamab	BCMA × CD3	Present case	Delayed-onset parkinsonism	Day 42 after treatment initiation	Clinically documented delayed-onset parkinsonism with neurocognitive symptoms, showing partial improvement after elranatamab discontinuation.

*BsAb*, bispecific antibody; *CNS*, central nervous system; *ICANS*, immune effector cell-associated neurotoxicity syndrome; *PMDA/JADER*, Pharmaceuticals and Medical Devices Agency/Japanese Adverse Drug Event Report database; *PML*, progressive multifocal leukoencephalopathy

^*^ PML represents an opportunistic CNS infection caused by JC virus reactivation and was therefore categorized separately from direct neurological toxicities

^**^ PMDA/JADER reports are spontaneous suspected adverse drug reaction reports and do not establish causality or incidence. Detailed clinical information, including timing of onset and clinical course was unavailable

 In our patient, parkinsonism developed several weeks after elranatamab initiation. No new medications other than elranatamab had been initiated during the relevant period, and the neurological symptoms partially improved after discontinuation of elranatamab. Brain MRI and DAT-SPECT showed no clinically significant abnormalities, and levodopa did not improve her symptoms. These findings made degenerative parkinsonism less likely and were consistent with features described in MNT-like neurological toxicity reported after BCMA-directed CAR-T cell therapy [13, 22–24]. Taken together, these clinical features support a possible association between elranatamab treatment and delayed-onset parkinsonism with neurocognitive symptoms.

The pathophysiological mechanisms of MNT remain unknown. Autopsy analyses of two patients who developed MNT after cilta-cel treatment revealed gliosis and T-cell infiltration localized to the basal ganglia [25]. Oekelon et al. reported BCMA expression in neurons and astrocytes of the caudate nucleus, and microarray data from healthy brains demonstrated BCMA RNA expression in the basal ganglia [22]. These observations imply that on-target, off-tumor recognition of BCMA within the CNS may underlie the pathogenesis of MNT associated with BCMA-targeting therapy. While BsAbs are generally considered to have limited blood–brain barrier (BBB) penetration [26, 27], cytokine-induced BBB disruption may facilitate lymphocyte infiltration into the CNS, resulting in neurotoxicity. Indeed, although the number of reports is limited, case reports describing CNS responses to elranatamab in patients with MM and CNS involvement after the development of CRS may further support the possibility that BsAbs can affect the brain parenchyma under conditions of cytokine release [26, 28].

No established treatment strategy has been defined for parkinsonism following BCMA-directed BsAb therapy. In CAR-T cell therapy-related MNT, corticosteroids and cyclophosphamide have been used, although responses are often limited, particularly in refractory cases [11, 12, 29–31]. In the present case, neurological symptoms began to improve approximately 3 weeks after discontinuation of elranatamab without specific immunosuppressive treatment. Nevertheless, the improvement was incomplete, and mild parkinsonism and cognitive impairment persisted. Improvement of neurological symptoms following treatment discontinuation alone may be attributable, at least in part, to the shorter half-life of elranatamab (approximately 22 days) [32].

In conclusion, we report a case of delayed-onset parkinsonism following elranatamab treatment, with clinical features resembling MNT previously described after BCMA-directed CAR-T cell therapy. Because this is a single case report, a causal relationship between elranatamab treatment and delayed-onset parkinsonism cannot be definitively established. Further accumulation of cases is essential to clarify the characteristics of this disorder and treatment responses and to establish diagnostic criteria and optimal management.

## Data Availability

No datasets were generated or analysed during the current study.
